# Exploring Douglas-Peucker Algorithm in the Detection of Epileptic Seizure from Multicategory EEG Signals

**DOI:** 10.1155/2019/5173589

**Published:** 2019-07-07

**Authors:** Roozbeh Zarei, Jing He, Siuly Siuly, Guangyan Huang, Yanchun Zhang

**Affiliations:** ^1^Ningbo Institute of Materials Technology & Engineering, Chinese Academy of Sciences, Ningbo, China; ^2^School of Information Technology, Deakin University, Melbourne, Australia; ^3^Institute of Information Technology, Nanjing University of Finance and Economics, Nanjing, China; ^4^Swinburne Data Science Research Institute, Swinburne University of Technology, Melbourne, Australia; ^5^Institute for Sustainable Industries & Liveable Cities, Victoria University, Melbourne, Australia

## Abstract

Discovering the concealed patterns of Electroencephalogram (EEG) signals is a crucial part in efficient detection of epileptic seizures. This study develops a new scheme based on Douglas-Peucker algorithm (DP) and principal component analysis (PCA) for extraction of representative and discriminatory information from epileptic EEG data. As the multichannel EEG signals are highly correlated and are in large volumes, the DP algorithm is applied to extract the most representative samples from EEG data. The PCA is utilised to produce uncorrelated variables and to reduce the dimensionality of the DP samples for better recognition. To verify the robustness of the proposed method, four machine learning techniques, random forest classifier (RF),* k*-nearest neighbour algorithm (*k*-NN), support vector machine (SVM), and decision tree classifier (DT), are employed on the obtained features. Furthermore, we assess the performance of the proposed methods by comparing it with some recently reported algorithms. The experimental results show that the DP technique effectively extracts the representative samples from EEG signals compressing up to over 47% sample points of EEG signals. The results also indicate that the proposed feature method with the RF classifier achieves the best performance and yields 99.85% of the overall classification accuracy (*OCA*). The proposed method outperforms the most recently reported methods in terms of* OCA* in the same epileptic EEG database.

## 1. Introduction

Epilepsy is one of the most common neurological disorders of the human brain that affects approximately 65 million people of the world [1]. It is characterised by unprovoked recurring seizures which are induced by abnormal and synchronous discharges of a group of neurons in the brain [2]. Although numerous molecular mechanisms underlying different forms of epilepsy have been identified, the etiology of majority of them cannot be explained by a simple defect altering ionic homeostasis [3]. Transient and unexpected electrical disturbances of the brain are recognised as the possible causatives for epileptic seizures. In the majority of cases, seizures occur unexpectedly, without a sign of warning to alert and prepare the person for an onset of a seizure. Such abrupt and uncontrollable nature of the disease can cause physical injury due to loss of motor control, loss of consciousness, or delayed reactivity during seizures. Impairment of consciousness can be life-threatening, especially if they occur while the person is driving, swimming, climbing heights, or alone. Electroencephalogram (EEG) is most commonly used technique for diagnosis of epileptic seizure in the medical community [4]. EEG record electrical activity along the scalp, via the placement on the scalp of multiple electrodes; it measures voltage fluctuations resulting from ionic current flows within the brain [5, 6]. Epileptic activity can create clear abnormalities on a standard EEG and leaves its signature on it [7]. Epileptic seizure activities in the brain commonly manifest spikes or spike wave complexes in EEG signals which are usually analysed visually by expert or neurologists [8, 9]. However, the visual scanning of EEG signal is very time-consuming and costly; it may be inaccurate, very complex, subject to judgement, and human error [10] as EEG signals contain a huge amount of data (in size and dimension). Therefore, there is an increasing need for developing automated epileptic seizure detection algorithms not only to alleviate the neurologist's burden of analysing long-term EEG signals but also to ensure a proper diagnosis and evaluation of neurological diseases.

In past two decades, several EEG signal processing techniques have been developed for automated epileptic seizure detection based on various feature extraction and classification techniques. The key challenge of any detection method is the extraction of the distinguishing features from EEG signals as it significantly affects the performance of the classifier. Representative characteristics or features extracted from EEG data can describe the key properties or morphologies of the signals for perfect detection of epileptic seizure [11]. As feature extraction is the most important part of detection process which plays key role in the performance of a classifier, this study aims to develop a new efficient feature extraction technique for the classification of epileptic seizure from EEG signals.

Several feature extraction methods have been applied in epileptic seizure detection, such as correlation [12], linear prediction error energy [13], fast Fourier transform (FFT) [14], wavelet transform [15–17], empirical mode decomposition (EMD) [18, 19], Lyapunov exponent [20], Correlation dimension [21], approximate entropy (ApEn) [22, 23], clustering technique [24], Sampling technique [10, 25], Complex network [6, 26, 27], and Optimum allocation [7, 28]. These feature extraction techniques can be grouped into four categories [29], namely, time-domain [12, 13], frequency domain [14], time-frequency domain [15, 16, 18, 19], and nonlinear methods [20–22]. Once features are extracted from EEG signals, a classifier is employed to differentiate between normal and epileptic EEG. Many classification methods have been proposed for seizure detection such as various types of artificial neural networks (ANNs) [30–32], support vector machine (SVM) [5, 12, 33, 34], Decision tree (DT) [35],* k*-nearest neighbour [36], and Random Forests (RF) [37].

Due to complex characteristics of EEG signals (e.g., nonstationary, aperiodic, and poor signal-to-noise ratio), sometimes it is very hard to achieve reasonable performance in the detection of epileptic seizure. Some of the existing feature extraction methods are not a good choice for obtaining characteristic features from nonstationary epileptic EEG data (e.g., Fourier transformation) [14, 38], and therefore most of their performances are limited regarding success rate and effectiveness [39, 40]. Moreover, the majority of the existing methods cannot appropriately handle large EEG data. Although most of the EEG recordings are multicategories in a real clinical application, most of the current methods are applied for binary EEG classification problems (Normal signal vs. ictal signal) [32, 41–45] and only a few methods focus on multiclass EEG classification [37, 39, 40, 46–48]. Considering these issues, this paper proposes a new feature extraction technique based on Douglas-Peucker algorithm (DP) and principal component analysis (PCA) for classification of multiclass EEG signals.

The DP [49] is the most well-known line simplification algorithm which is widely used in cartographic and computer graphic applications to reduce the complexity and storage requirements of curves by removing curve's no-characteristic points and extracting characteristic points [50–53]. It is also applied in biomedical applications such as Electroencephalogram (ECG) signals compression [54–56]. The main theme of this algorithm is to shorten a line by detecting and preserving the most significant points of a line while neglecting less important points. Although the DP technique has a high capability to represent the original patterns of time series data and reduce the size of data, it has not been considered before for epileptic detection in the EEG signal analysis to the best of author's knowledge. Thus this study introduces for the first time the idea of using the DP methods for extracting representative sampling points from huge amount of raw EEG data.

The main aim of this research is to develop a novel feature extraction technique for detection of epileptic seizure from multicategory EEG signal for properly handling big size EEG data. Moreover, this paper investigates the effectiveness of DP algorithm in the detection of epileptic seizure from EEG data and also discovers an effective classifier for the proposed features. In the proposed methodology, first the nonstationary epileptic EEG signals are partitioned into some nonoverlapping segments (called* Segm*) to make them stationary (discussed in detail in Section 3.1.1). Then the DP algorithm is effectively employed to extract representative sampling points from each* Segm* and also to reduce the size of each* Segm* by removing redundant points. At the next stage, the PCA is used to reduce the dimensionality of DP data and also to produce uncorrelated variables which are considered as features, denoted as DP_PCA feature set. In order to select an efficient classifier for DP_PCA feature set, this study employs four popular machine learning techniques namely, RF,* k*-nearest neighbour algorithm (*k*-NN), SVM, and DT on the extracted features. To evaluate the consistency and performance of the proposed methods, tenfold cross-validation is applied to create training and testing set. The performance of each method is evaluated by sensitivity (*Se*), specificity (*Sp*), overall classification accuracy (*OCA*), false positive rate (*FPR*), kappa statistic, and receiver operating characteristic (*ROC*) curve area. In order to further evaluate the performances, the proposed method is compared with other six existing algorithms. The experiment results show that the RF classifier is the best classifier for DP_PCA feature set compared to other three classifiers. The results also indicate that the proposed method outperforms the existing methods [37, 39, 40, 46–48] regarding* Se*,* Sp*, and* OCA*.

The rest of the paper is organized as follows: in Section 2, we describe the prior studies in multiclass EEG signals classification.  Section 3 presents the methodology of the proposed method.  Section 3 also describes the experimental data and implementation.  Section 4 discusses the experimental results and discussions. Finally, Section 5 draws the conclusion for this paper.

## 2. Previous Work

In the last decade, various methods have been proposed for the classification of EEG signals [1, 2, 8, 9, 12–16, 22, 29, 36, 57–60]. However, only a few approaches have dealt with multiclass EEG classification problems [37, 39, 40, 46–48]. For comparative reasons, the most recent and relevant studies dealing with multiclass EEG classification problems on a benchmark epileptic EEG dataset [61, 62] are reviewed.

Most recently, Emigdio et al. [37] developed a method based on Holderian regularity and the Matching Pursuit (MP) algorithm for feature extraction in the epileptic EEG signal classification. The feature sets were constructed by combining features extracted from EEG signals through regularity analysis, the MP algorithm and simple time-domain statistical analysis. These feature sets were then fed to a Random Forests classifier for classification of epileptic states. The performance of the method was tested on the Bonn data set [61, 62] considering different classification problems (binary classification problems and multiclass classification problems). The results showed that the overall classification accuracy was 97.6% for the five-class classification problem.

Murugavel and Ramakrishnan [48] introduced an approach based on a hierarchical multiclass SVM (H-MSVM) with extreme learning machine (ELM) as the kernel for the classification of epileptic EEG signals. The wavelet transform was used to decomposed the EEG data into six subbands and then six features such as largest Lyapunov exponent, statistical values, and approximate entropy were extracted from each subband. The extracted features were employed as the input to the classifier. The artificial neural network (ANN) and multiclass SVM were also utilised to identify the five-category EEG signals. The experimental results showed that the H-MSVM classifier with ELM kernel yielded a better performance regarding classification accuracy and computation complexity compared to the ANN and SVM classifiers. The H-MSVM achieved an overall classification accuracy of 94%.

Ubeyli [47] reported a method based on Lyapunov exponents and a probabilistic neural network (PNN) classifier for classification of EEG signals. The Lyapunov exponents were obtained from each EEG signal using Jacobi-based algorithms and considered as feature vectors. The statistic over the Lyapunov exponents was used to reduce the dimensionality of the extracted feature vectors. The selected features were fed to the PNN and multilayer perceptron neural network (MLPNN) classifiers. The classification results show that the PNN with Lyapunov exponents features achieved an overall classification accuracy of 98.05% while the MLPNN produced a 92.20% accuracy rate.

Ubeyli [46] presented automated diagnostic systems combined with spectral analysis techniques for classification of EEG signals. Eigenvector methods were used to calculate the wavelet coefficients and power spectral density (PSD) values which considered as features. The selected features then were fed to seven classification algorithms: SVM, PNN, mixture of experts (ME), modified mixture of experts (MME), recurrent neural networks (RNN), MLPNN, and combined neural networks (CNN). The experimental results showed that the SVM and MME classifiers achieved better performance compared to other five classifiers. The classification accuracy for the SVM, MME, PNN, ME, RNN, CNN and MLPNN classifiers with the obtained features were 99.20%, 98.68%, 95.30%, 95%, 94.85%, 93.48%, and 90.48%, respectively.

Ubeyli [39] developed a method based on multiclass SVMs with the error correcting output codes (ECOC) and eigenvector methods for the classification of EEG signals. The PSD values of the EEG signals were obtained using three different eigenvector methods such as the MUSIC [63], Pisarenko [64], and minimum-norm [65]. The statistics over the set of the power levels of the PSDs were considered as features and fed to the multiclass SVMs. The MLPNN classifier was also applied to the same feature set. The total classification accuracy obtained by SVM with the ECOC and the MLPNN was 99.30% and 92.90%, respectively.

Guler and Ubeyli [40] proposed the multiclass SVM with the ECOC for the classification of multiclass EEG signals. They also tested the probabilistic neural network (PNN) and multilayer perceptron neural network (MLPNN) classifiers on the same epileptic EEG data. The wavelet coefficients and Lyapunov exponents were used to extract features from the EEG data. The extracted features were employed as the input of the three classifiers. The results showed that the multiclass SVM classifier achieved better performance than the other two classifiers. The total classification accuracy for the SVM, PNN, and MLPNN was 99.28%, 98.05%, and 93.63%, respectively.

## 3. Methods and Materials

### 3.1. Proposed Approach

**Figure 1:**
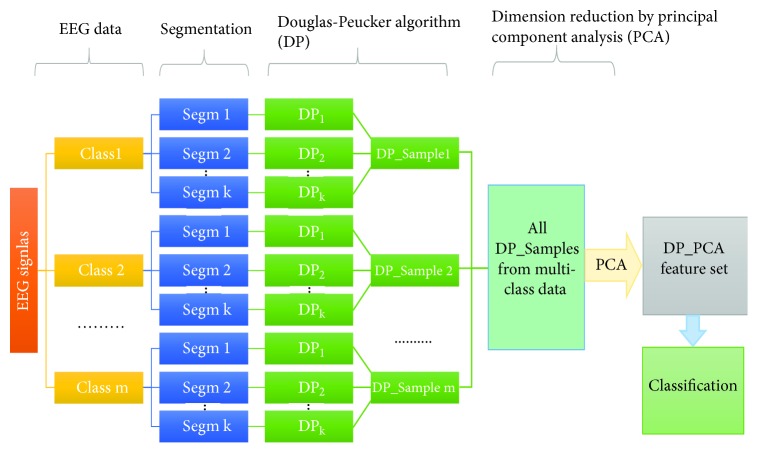
Block diagram of the proposed method for the classification of epileptic EEG signals.

The paper introduces a novel method based on DP in the multiclass EEG signal classification. In this study, the DP approach is developed to select representative samples from the original EEG signals that reflect an entire database. Next, The PCA is used to reduce the dimension of the obtained DP sample set which is considered as a feature set. Finally, the extracted features are tested by four machine learning methods, including RF,* k*-NN, SVM, and DT. As shown in Figure 1, the entire process of proposed method is divided into five major parts: data segmentation, Douglas-Peucker algorithm, dimension reduction by PCA, DP_PCA feature set, and the classification part by the RF,* k*-NN, DT, and SVM. A detailed description of these five parts is provided in the following sections.

#### 3.1.1. Data Segmentation

**Figure 2:**
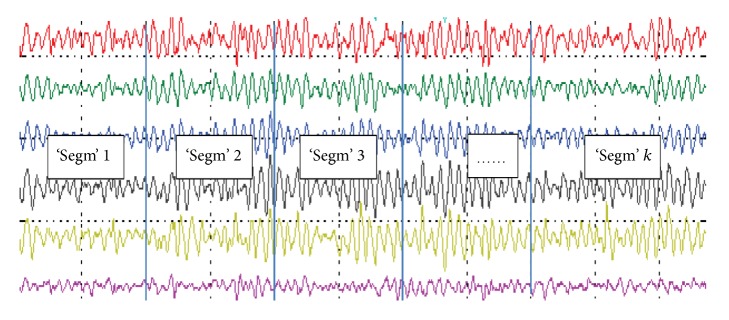
An example of determining* Segms* from an EEG signals of a class.

Most of the EEG signal processing methods require stationarity of the signals. Although EEG signal may not be stationary, usually smaller windows or parts of those signals will exhibit stationarity [7]. An EEG signal is stationary for a small amount of time. That is the reason the recorded EEG signals of every class are split into several nonoverlapping segments based on a particular time period to properly account for possible stationarities. Hence the EEG signals of each class are segmented into some fixed-size nonoverlapping time windows (called* ‘Segm'*) to obtain representative values of a specific time period. Each* Segm* consists of EEG channel data within a time window.  Figure 2 illustrates an example of determining the segments* Segms* in an EEG signal of a class. It is worthwhile to mention that the number of* Segms* (*k*) is determined empirically over time for any experiment design.

#### 3.1.2. Douglas-Peucker Algorithm

**Figure 3:**
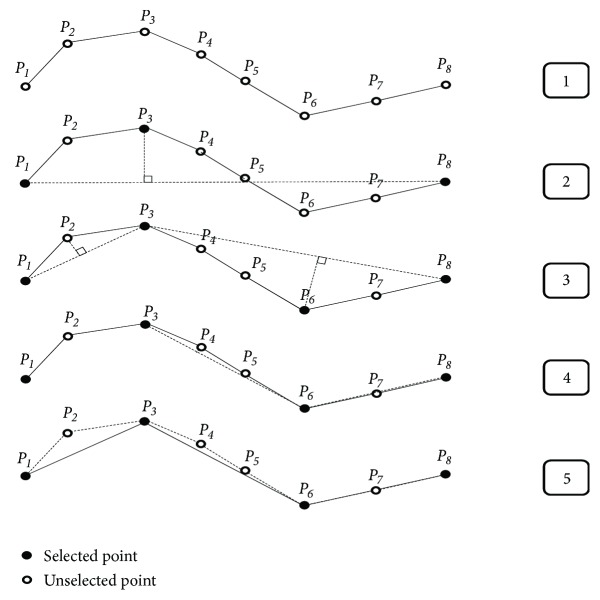
An example of DP sample point extraction.

The DP [49] is one of the most popular methods for line (trajectory) simplification. The algorithm simplifies a line by detecting and preserving the most significant points of a line while neglecting less important points. In this study, the DP technique is used to extract the representative samples from different* ‘Segms'*. Let the data series (trajectory)* S* be described by the set of* N* points <*P*_1_, *P*_2_,…, *P*_*N*_>. The main idea of DP algorithm is to determine a new data series with fewer and most significant points without deviating from the original data series by at most a simplification tolerance *ϵ*. As an initial step of DP, the algorithm approximates the data series* S* with a line segment $\bar{P_{1}P_{N}}$ constructed from the first to the last data point. Then it calculates the perpendicular Euclidean distance between each intermediate data point and the line segment $\bar{P_{1}P_{N}}$ and retains the point *P*_*i*_ which has the maximum distance *D*_*max*_. The algorithm compares *D*_*max*_ with the given simplification tolerance *ϵ*. If the maximum distance *D*_*max*_ is less than the simplification tolerance *ϵ*, the algorithm removes all intermediate points in data series. Otherwise, it uses data point *P*_*i*_ to split the data series to two subseries <*P*_1_, *P*_2_,…, *P*_*i*_> and <*P*_*i*_, *P*_*i*+1_,…, *P*_*N*_> and recursively repeats the procedure for each subseries. The DP algorithm terminates when the *D*_*max*_ in a subseries is lower than the simplification tolerance *ϵ* or the subseries contains only two data points.  Figure 3 illustrates an example of DP sample point extraction. The original data series contain eight points (*P*_1_*P*_8_). The distances from the points *P*_1_*P*_8_ to the line segment $\bar{P_{1}P_{8}}$ are first computed. Since the maximum distance *D*_*max*_ at point *P*_3_ exceeds the given simplification tolerance *ϵ*, the data series are divided at this point into two subseries (step 2 in Figure 3). In the left subseries, the distance from *P*_2_ to the line segment $\bar{P_{1}P_{3}}$ is lower than *ϵ* value, so the point *P*_2_ is ignored. In the right subseries, the distance from the point *P*_6_ to the line segment $\bar{P_{3}P_{8}}$ also exceeds the simplification tolerance *ϵ*, hence a new split is performed at the point *P*_6_, and the process is repeated for each part, respectively.  Figure 3 shows that the original data series having 8 points finally becomes a 4-points data series after this process.

The value of simplification tolerance *ϵ* determines the degree of simplification. Therefore, it is an important task in DP algorithm to determine the most significant *ϵ* value. Choosing a small value of *ϵ* will produce a minimally simplified data series (i.e., only a few redundant data points will be removed from data series) while selecting a large one will provide a highly simplified data series that might lead to losing some of the significant points from the data series. The following formula (1) is used to calculate the most significant *ϵ* value for each data series.

(1) $$\begin{array}{c} \varepsilon =\frac{T}{100}\times \left(\;\overset{N-1}{\underset{i=1}{\sum }}D\left(\;P_{i+1},P_{i}\;\right)\;\right) \end{array}$$

where* N* is the number of points in data series; *D*(*P*_*i*+1_, *P*_*i*_) is the Euclidean distance between two points *p*_*i*+1_ and *p*_*i*_; ∑_*i*=1_^*N*−1^*D*(*P*_*i*+1_, *P*_*i*_) is the overall distance of the data series; and* T* is a real number which is determined empirically. The value of *ϵ* is changed as different percentages of the overall distance of the data series by setting different values for* T*.

As shown in Figure 1, the DP process consists of the following steps to extract the representative samples from various* Segms*.

**Step 1 .**

Consider all the channels of the EEG data of a class.

**Step 2 .**

The EEG data of that class is split into* k Segms* considering a particular time period. Suppose the sizes of the* Segms* are *N*_1_, *N*_2_,…, *N*_*k*_, respectively.

**Step 3 .**

The overall distance of each* Segm* is calculated. Then by setting the* T* value, the value of simplification tolerance *ϵ* for each* Segm* is calculated using (1).

**Step 4 .**

The representative samples from each* Segm* are extracted using DP algorithm. Let *n*_1_, *n*_2_,…, *n*_*k*_ be the sizes of samples obtained from the* Segms* whose sizes are *N*_1_, *N*_2_,…, *N*_*k*_, respectively. The representative samples selected from each* Segm* in a class make a vector set denoted as DP_Sample as shown in Figure 1.

**Step 5 .**

The vector sets of all classes construct a matrix (denoted as DP_samples set) that is used as input to the PCA, as discussed in the next section.

#### 3.1.3. Dimension Reduction by PCA

The PCA is a well-known statistical method for feature extraction and dimensionality reduction [66–68]. It uses an orthogonal transformation to convert a set of observations of possibly correlated variables into a smaller set of uncorrelated variables called principal components (PC). These components represent the most important linear characteristics of the data. The multichannel EEG signals recorded from different scalp sites are highly correlated. They contain a large amount of redundant information. Therefore, it would be useful to remove this redundant information by converting the EEG signals into a set of new linearly uncorrelated variables (i.e., the PC space) and utilise these new variables as features for better classification of EEG signals. In this paper, the PCA is used to reduce the dimensionality of the DP_Samples set and also to obtain EEG features for classification of epileptic EEG signals.

Let *DP*_*samples*  *set* = [*x*_1_^*T*^; *x*_2_^*T*^; …; *x*_*n*_^*T*^] ∈ *R*^*n*×*p*^ where each row *x*_*i*_ represents a data point in a* p*-dimensional space (considering a* p*-channel EEG signals as a* p*-dimensional space) and* n* is the number of the points selected by DP. PCA can be formulated as the following optimisation problem:

(2) $$\begin{array}{c} \underset{U\in R^{p\times k},\left\|U\right\|=I}{max}\overset{n}{\underset{i=1}{\sum }}U^{T}\left(\;x_{i}-\mu \;\right){\left(\;x_{i}-\mu \;\right)}^{T}U \end{array}$$

where** U** is a matrix consisting of* q* dominant eigenvectors. This problem can be solved by deriving an eigenvalue decomposition problem of the covariance matrix.

(3) $$\begin{array}{c} {\hat{C}}_{x}U=U\Lambda \end{array}$$

where

(4) $$\begin{array}{c} {\hat{C}}_{x}=\frac{1}{n}\overset{n}{\underset{i=1}{\sum }}\left(\;x_{i}-\mu \;\right){\left(\;x_{i}-\mu \;\right)}^{T} \end{array}$$

is the covariance matrix, *μ* is the global mean defined as *μ* = (1/*n*)∑_*i*=1_^*n*^*x*_*i*_, Λ_*i*_  (*i* = 1,2,…, *p*) are the eigenvalues and they are sorted in descending order, and *U*_*i*_  (*i* = 1,2,…, *p*) are the corresponding eigenvectors. In order to reduce the dimensionality of the DP_Samples set, only the first* q* eigenvector (*q* ≤ *p*) which corresponds to the* q* largest eigenvalues is selected by the following equation to represent the DP_Samples set.

(5) $$\begin{array}{c} \frac{\sum_{i=1}^{q}\Lambda_{i}}{\sum_{i=1}^{p}\Lambda_{i}}\geq \sigma \end{array}$$

For a given precision parameter *σ* (considering *σ*=90% in this study), the matrix ** U** consisting of* q* dominant eigenvectors is constructed and the* q*-dimensional feature set denoted as DP_PCA feature set is computed as follows:

(6) $$\begin{array}{c} DP\_PCA=\left(\;DP\_Samples\;\;set\;\right)U \end{array}$$

#### 3.1.4. DP_PCA Feature Set

The new feature set donated as DP_PCA feature set is generated by reducing the dimensionality of the DP_Samples set using PCA method as discussed in Section 3.1.3. This feature vector set is divided into a training set and a testing set using a tenfold cross-validation method, which is discussed in Section 3.3. As shown in Figure 1, this feature set is fed to each of the four classifiers discussed in the next section.

#### 3.1.5. Classification by the RF, KNN, SVM, and DT

This study considers four classifiers: RF,* k*-NN, DT, and SVM for testing the performance of the proposed feature extraction method. A brief explanation of these classification methods is provided in the following sections.

**Figure 4:**
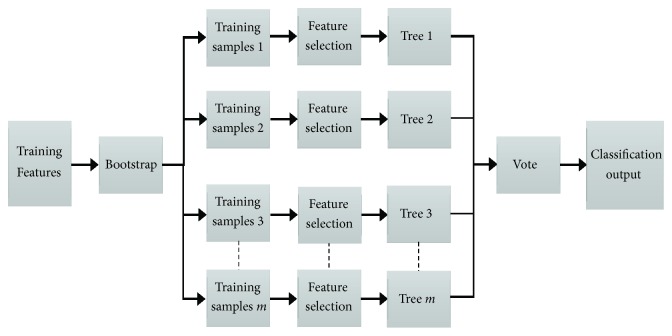
The structure of random forest classifier.

*Random Forest*. The RF is an ensemble learning technique developed by Breiman [69]. It consists of many individual classification trees, where each tree is constructed using a tree classification by selecting a random subset of input features and a different bootstrap sample from the training data. The RF aggregates the results of all classification trees to classify new samples. Each tree casts a unit vote at the input data and then the forest selects the class with the most votes for the input data.  Figure 4 illustrates the structure of random forest classifier.

The RF algorithm proceeds as follows:

1. From the training data set,* m* training subsets are generated using the bootstrapping technique (randomly sampling with replacement). Each training subset has the same size as the training data set and contains approximately one-third of the samples of the training data set.
2. For each training subsets, a decision tree is built with the following criteria: at each node in building a decision tree, a random number of f features are selected from the* F* input features (*f* ≪ *F*) and the best split (e.g., the largest Gini measure) among these* f* features is used to divide the node. The tree is grown to the maximum size with no pruning. The tree growing algorithm used in RF is random trees.
3. The* m* trees are combined into an RF ensemble and use a majority voting scheme to predict the class of new data by evaluating votes from each tree.

*K-Nearest Neighbour Algorithm*. The* k*-NN is a supervised learning algorithm for classifying objects based on closest training observations in the feature space [66, 70]. Although the* k*-NN is the simplest algorithm among all machine learning algorithms, it can still yield high performance, without a priori assumptions about the distributions from which the training samples are drawn [66]. Given a query vector *x*_0_ and a set of* N* labelled instances {x_*i*_, *y*_*i*_}_1_^*N*^, the aim of the classifier is to identify the class label of *x*_0_ on the predefined* P* classes. The* k*-NN classification algorithm tries to find the k-nearest neighbours of *x*_0_, and uses a majority vote to determine the class label of *x*_0_. Without prior knowledge, the* k*-NN classifier usually applies Euclidean distances as the distance metric [71]. A detailed discussion of this method can be found in [66, 70, 72].

*Support Vector Machine*. The SVM is a machine learning algorithm based on statistical learning theory and structural risk minimisation principle presented by Vapnik [73]. The main idea of SVM is to map the input data into a higher dimensional space and then determines an optimal separating hyperplane between the two classes of data in the transformed space [74, 75]. For nonlinear classifier models, when the data are not linearly separable, SVMs map inseparable input data into a high-dimensional space by constructing a linear kernel function to make the input data linearly separable in new space and allows better fitting of the hyperplane to the input dataset. Although the SVM is originally designed as a two-class classifier, some methods have been proposed to extend the application of SVM to multiclass classifications. One common used procedure in practice is to employ a set of pairwise classifiers, based on one-against-one decomposition [75]. The decision function of binary SVM classifier can be expressed as follows:

(7) $$\begin{array}{c} f\left(\;x\;\right)=sgn\left(\;\overset{s}{\underset{i=1}{\sum }}y_{i}\alpha_{i}k\left(\;x_{i},x\;\right)+b\;\right);\;0<\alpha_{i}<C \end{array}$$

where* sgn* is the signum function, *k*(*x*_*i*_, *x*) is kernel function, and* b* is the bias of the training samples. There are several kernel functions such as linear kernel, polynomial kernel, RBF kernel, and sigmoid kernel. In this paper, the polynomial kernel is considered as the best kernel function for identifying multicategories EEG signals as it was found to give the best classification performance.

The regularisation parameter* C* is used to control the trade-off between training error and model complexity and can be calculated as follows:

(8) $$\begin{array}{c} C=\frac{N}{\sum_{i=1}^{N}K\left(x_{i},x\right)} \end{array}$$

where* N* is the size of the training set.

In the multiclass classification problem, the SVMs work by using a collection of decision functions *f*_*kl*_. The class decision can be obtained by the following formula [75]:

(9) $$\begin{array}{c} f_{k}\left(\;x\;\right)=\overset{n}{\underset{i=1}{\sum }}sgn\left(\;f_{kl}\left(\;x\;\right)\;\right) \end{array}$$

where* kl* indicates each pair of classes selected from separated target classes and* n* is the number of separated target classes. The algorithm proceeds as follows: it assigns a label to the class: arg max *f*_*k*_(*x*), (*k = 1, 2,..., n*). The pairwise classification then converts the* n*-class classification problem into* n(n1)/2* two-class problems which cover all pairs of classes. An overview of SVM classifier can be found in [73–75].

*J48 Decision Tree*. J48 decision tree is an implementation of the C4.5 algorithm [76] in the WEKA (The Waikato Environment for Knowledge Analysis) [77, 78]. C4.5 is an extension of the ID3 algorithm. It uses the top-down construction technique to recursively split the data set into smaller subsets based on the value of an attribute [76, 79]. This classifier builds a decision tree for the given dataset using the concept of information entropy. In a decision tree, each attribute can be used to make a decision by splitting the data into smaller subsets. At each node of the tree, the algorithm evaluates each attribute of the data for dividing the data into smaller subsets and chooses the attribute that gives the highest information gain. Once an attribute is selected, the data set is split into subsets, and the splitting process is repeated for each subset until further splitting is not gainful. In the resulting tree structure; each inner node in the tree corresponds to one of the input attributes, each branch represents a value or range of values of that attribute, and each leaf accounts for a classification.

### 3.2. The Epileptic EEG Data

**Figure 5:**
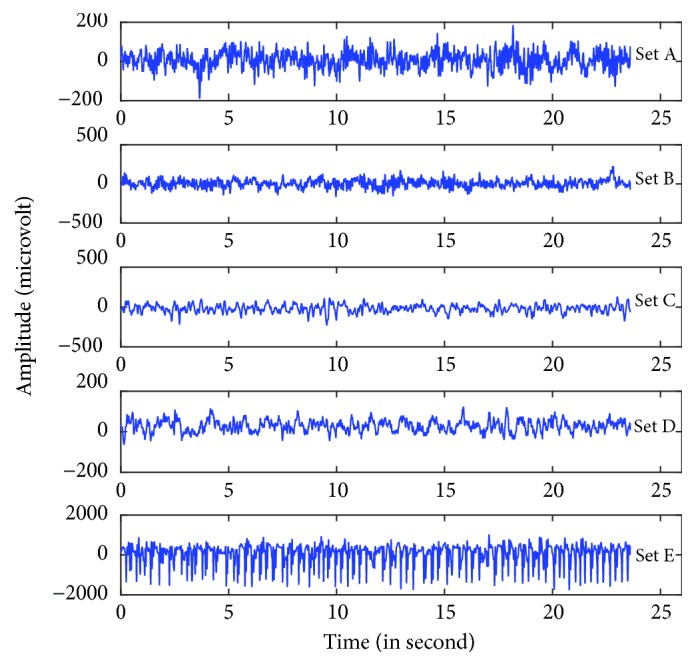
Example of five different sets of EEG signals taken from different subjects. The figure is reproduced from K. Revett et al. [81] (under the Creative Commons Attribution License/public domain).

The epileptic EEG data used in this work is obtained from publicly available EEG database of Department of Epileptology, University of Bonn, Germany [61, 62]. The whole database contains five subsets denoted as Sets A, B, C, D, and E. Each subset is containing 100 single-channel EEG signals with a duration of 23.6 s. The subsets A and B are recorded extracranially, whereas subsets C, D, and E are recorded intracranially. Set A and Set B were collected from five healthy volunteers with eyes open and eyes closed, respectively. Sets C and D were collected from five epileptic patients during interictal periods. Set C was recorded from the hippocampal formation on the opposite side of the epileptogenic zone while Set D was recorded from the epileptogenic zone. Set E was collected from all of the recording zones in Sets C and D during seizure activity (ictal periods). All EEG recordings were recorded using a 128-channel amplifier system with a sampling rate of 173.61 Hz and 12-bit A/D resolution. Signals were filtered using a 0.53–40 Hz (12 dB/octave) band pass filter, and artifacts such as muscle and eye movements were removed by visual inspection. A summary description of the five set EEG data is provided in Table 1. Exemplary EEG time series from each of the five classes (Set A-Set E) are shown in Figure 5

### 3.3. Implementation

**Figure 6:**
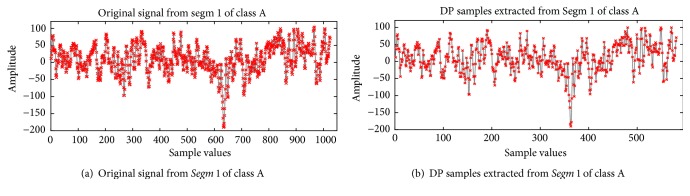
The typical results of DP for the healthy subject.

**Figure 7:**
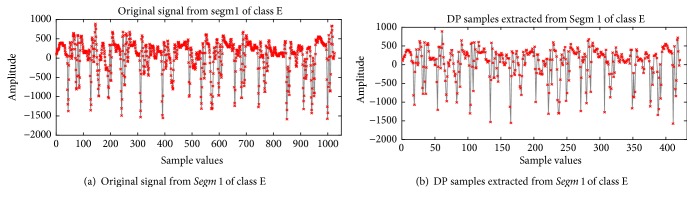
The typical results of DP for the epileptic patient.

**Figure 8:**
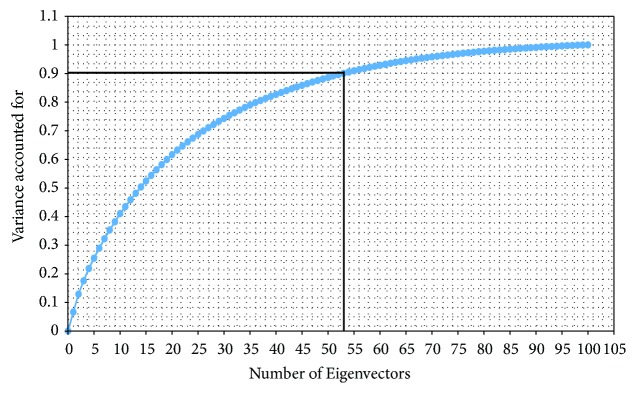
The cumulative eigenvalues for all 100 eigenvectors.

This section presents the implementation of the proposed method on the epileptic EEG data [61, 62]. As discussed in Section 3.2, the complete dataset contains five sets (denoted as A, B, C, D, and E), each containing 100 channels data of 23.6 s. Each channel consists of 4096 data samples. The implementation of the proposed method comprises five steps as follow:

1. Each class data is segmented into four* Segms* (*K*=4), each containing 100 channels data of 5.9 s. As each channel consists of 4096 data samples, the sizes of the four* Segms* are *N*_1_=1024, *N*_2_=1024, *N*_3_=1024, and *N*_4_=1025, respectively.
2. To determine the value of simplification tolerance *ϵ*, the overall distance of each of the four* Segms* in each class are calculated, and then the value of* T* is changed from 0.01 to 0.1 with step size 0.01 in (1) to identify the most significant *ϵ* value for each* Segm*. From the experiment, it is considered that* T*=0.06, and then the value of *ϵ* for each* Segm* is calculated using (1).  Table 2 presents the obtained value of *ϵ* for each* Segm* in each of the five classes. From Table 2, it is observed that the values of *ϵ* are not equal due to the differences in the overall distance of* Segms*.
3. Using the obtained value of *ϵ* shown in Table 2, the representative samples from each* Segm* are extracted using the DP algorithm. Figures 6 and 7 show typical results of DP for the healthy subject (class A) and the epileptic patient (class E), respectively. In Figures 6 and 7, the first* Segm* of class A and class E is considered, respectively. It can be seen from Figures 6 and 7 that the DP samples can effectively represent the original signals with fewer points which indicate the ability of DP to select most significant points from each signal.
4. Table 3 provides the number of the representative samples chosen by DP for each* Segm* in each of the five classes. It can be seen from Table 3 that the number of the representative samples for each* Segm* is not equal; e.g., in Set A (Class 1), the total number of 581, 612, 599, and 562 samples is selected by DP from* Segm* 1,* Segm* 2,* Segm* 3, and* Segm* 4, respectively. The total number of the representative samples is 2354 for Set A.
5. The representative samples selected from all* Segms* in a class create a vector set denoted as DP_Sample as shown in Figure 1. For example, the selected representative samples from each of the four* Segms* of class 1 create a DP_Sample 1 vector. The DP_Sample 1 is constructed as 581, 612, 599, and 562 which contains all 2354 selected samples in class 1. The vector sets are created similarly: DP_Sample 2, DP_Sample 3, DP_Sample 4, DP_Sample 5 from class 2, class 3, class 4, and class 5, respectively. All DP_Samples from the five-class EEG data construct a matrix denoted as DP_sample set that is used as an input to the PCA. The DP_sample set contains all 10755 selected samples from five classes (2354 for class 1, 2008 for class 2, 2237 for class 3, 2398 for class 4, 1758 for class 5). It can be seen from Table 3 that the DP reduces the data samples size of the five class from 20480 sample points to 10755 sample point (47.49% sample reduction). Here note that each sample has 100 dimensions as each class contains 100 channels of EEG data. Therefore, the DP_sample set consists of 10755 samples of 100 dimensions.
6. The PCA is applied to reduce the dimensionality of the DP_ Sample set. Only the first q eigenvectors are selected to represent the DP_sample set based on the accumulation of their respective eigenvalues exceed 90% of total sum of eigenvalues (see (5)).  Figure 8 illustrates the cumulative eigenvalues for all 100 eigenvectors. It is observed that the accumulation of the first 53 eigenvalues exceeds 90% of total sum of eigenvalues. Therefore, only the first 53 eigenvectors are considered for obtaining the DP_PCA feature set. The obtained DP_PCA feature set contains 10755 samples of 53 dimensions.
7. The DP_PCA feature set is divided into a training set and a testing set using a 10-fold cross-validation method to evaluate the performances of the proposed methods. The DP_PCA feature set is split into ten mutually exclusive subsets (10-folds) of approximately equal sizes. Training and testing are performed ten times. Each time, one of the folds is used as a testing set and the remaining nine folds are combined into a set for training.

In this research, the performances of the proposed methods are evaluated based on different statistical measures, such as* Se*,* OCA*,* FPR*, kappa statistic, and* ROC* curve area. Their formulas are given below:

(10) Sei=No.  of  true  positive  decisions  in  class  iNo.  of  actual  positive  cases  in  class  i×100,i=1,2,3,4,5

(11) OCA=No.  of  correct  decisions  for  all  classesTotal  no.  of  cases  for  all  classes×100

(12) FPRi=No.  of  negative  cases  that  are  detected  as  posative  cases  in  ith  classesTotal  no.  of  negative  cases×100

(13) Kappa=Po−Pe1−Pe

where *P*_*o*_ denotes the overall observed agreement between the classifier and the true classes, and *P*_*e*_ represents the expected proportion of agreement. Besides, the Area Under the* ROC* Curve (AUC) is measured to compare the overall performance of the classifiers. The* ROC* curve is obtained by plotting the sensitivity versus false positive rates [47].

## 4. Results and Discussions

This section presents the experimental results of the proposed methods on the epileptic EEG datasets. First, the effectiveness of each of the four mentioned classifiers is evaluated on the DP_PCA feature set to select the most appropriate classifier as discussed in Section 4.1. Then a comparison between the proposed method and six existing methods is provided in Section 4.2. All mathematical calculations are carried out in MATLAB R signal processing tool (version 7.11, R2010b). The classification executions for all four classifiers: RF,* k*-NN, SVM, and J48 classifiers are executed in WEKA machine learning toolkit [77, 78]. The LIBSVM tools (version 3.2) [80] is used in WEKA for the SVM classification. It is worth mentioning that the default parameter values for each classifier in WEKA are used as there are no specific guidelines for selecting these parameters.

### 4.1. Classification Results for Each Classifier

As mentioned before, four machine learning methods, RF,* k*-NN, SVM, and J48 classifiers, are tested for detection of the multiclass EEG signals.  Table 4 presents the classification results of all classifiers on the DP_PCA feature set. The performance results are given by averaging over the results of the 10-fold cross-validation test and expressed as the mean ± standard deviation. As shown in Table 4, the RF classifier achieves the highest classification performance in terms of average classification accuracy which is 99.85%. The* k*-NN classifier stands at the second position and the SVM classifier achieves the third position with the average classification accuracy of 98.31% and 96.11%, respectively. The J48 classifier yields the lowest average classification accuracy among all tested methods. It can be seen from Table 4 that the RF classifier produces the best performance in terms of sensitivity among all classifiers and obtains the sensitivity rate of 99.79% for Set A, 99.85% for Set B, 99.96% for Set C, 99.71% for Set D, and 100% for Set E. It is observed that both* k*-NN and SVM classifiers yield high sensitivity rates in Sets A, B, C, and D, but they failed to correctly classify the epileptic patient during seizure activity class (Set E).  Table 4 also shows that the standard deviation for every classifier is very low which indicates the consistency of the mentioned classifiers for the DP_PCA features set.

**Figure 9:**
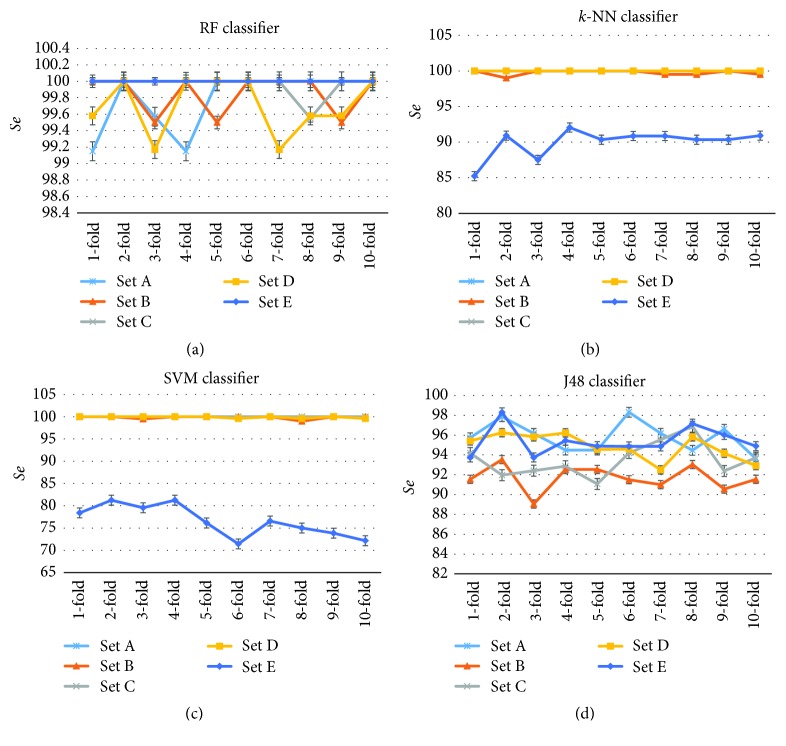
Individual classification performances of each of the ten folds in each class for the proposed classifiers: (a) RF, (b) KNN, (c) SVM, and (d) J48.

**Figure 10:**
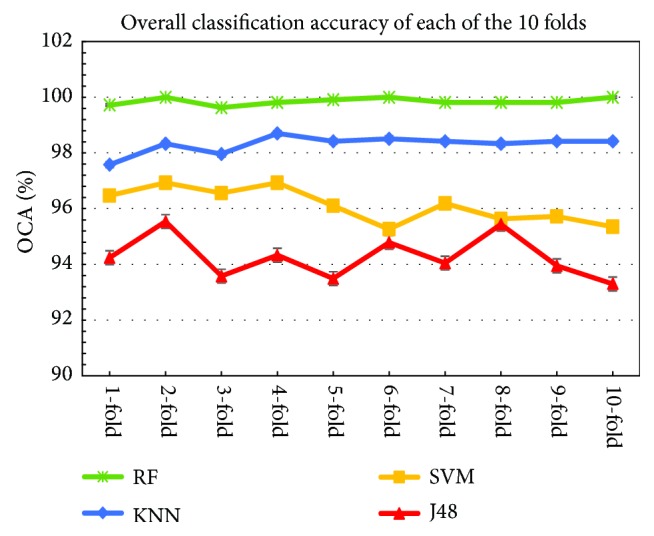
The overall classification accuracy (*OCA*) in each of the ten folds.

To provide more detailed information about how the 10-fold cross-validation system produces the classification performance regarding sensitivity and accuracy in each of the ten folds for each of the four classifiers, the classification results in each of the ten folds are provided in Figures 9 and 10.  Figure 9 illustrates the classification results in terms of the* Se* in each of the ten folds in each class (Set) for the reported classifiers and Figure 10 shows the classification results for all classifiers in terms of the* OCA* in each of the ten folds. The error bars in these figures represent the standard errors.

Figure 9 presents the patterns of the* Se* for each class. From Figure 9(a), little fluctuation is noted in the* Se* patterns among the ten folds in each of the five classes for the RF classifier. These results indicate the stability and robustness of the RF classifier. From Figures 9(b) and 9(c), it is seen that the* Se* patterns for Set A, Set B, Set C, Set D are almost similar but the patterns for Set E is different and dramatically lower than the other patterns for both* k*-NN and SVM classifiers. This indicates the weakness of the* k*-NN and SVM classifiers for detecting the epileptic signals during seizure activity class (Set E).  Figure 9(d) shows that the J48 classifier produces similar* Se* patterns for all Sets. It can be seen from Figure 9 that the fluctuations in the* Se* patterns among the different folds are negligible in each class for all classifiers. These results demonstrate the consistency of the classification methods.

Figure 10 shows the overall classification accuracies against each of the 10-folds for all classifiers. As can be seen from Figure 10, the RF classifier yields the best performance for each of the 10-folds compared to the* k*-NN, SVM and the J48 classifiers. It is observed that the fluctuations of the performance of the RF classifier are smaller among the different folds compared to other classifiers, indicating the stability of the RF classifier for the DP_PCA features Set. This figure also shows that the* k*-NN classifier produces a better performance than both SVM and J48 classifiers in each of the 10-folds. The lowest performance is obtained by the J48 classifier in each of the 10-folds.

Table 5 provides the* FPR* for the four classifiers in each of the ten folds for Set A, Set B, Set C, Set D, and Set E. In Table 5, the overall results of each class (Set) are also reported in terms of mean ± standard deviation of the* FPR* over a 10-fold cross-validation. It is observed from Table 5 that the RF classifier produces an overall* FPR* of 0.04% for Set A, 0.03% for Set B, 0.11% for Set C, 0.01% for Set D, and 0.00% for Set E, while these values are 0.18%, 1.09%, 0.36%, 0.50%, and 0.00%, respectively, for the* k*-NN classifier; and 1.06%, 1.63%, 1.14%, 1.07%, and 0.00%, respectively, for the SVM classifier; and 1.18%, 1.81%, 2.20%, 1.75%, and 0.29%, respectively, for the J48 classifier. The results show that in most cases the* FPRs* are zero in each of the folds in all classes for the RF classifier. It is also observed that the overall* FPRs* of the RF classifier are lower than those of the* k*-NN, the SVM, and the J48 classifiers in all classes.

**Figure 11:**
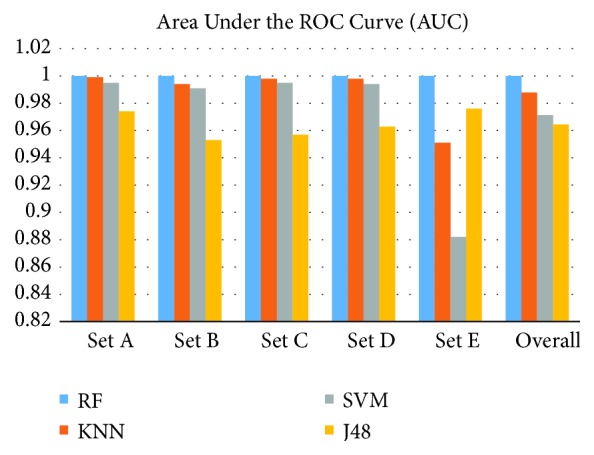
The AUC for the proposed classifiers.

**Figure 12:**
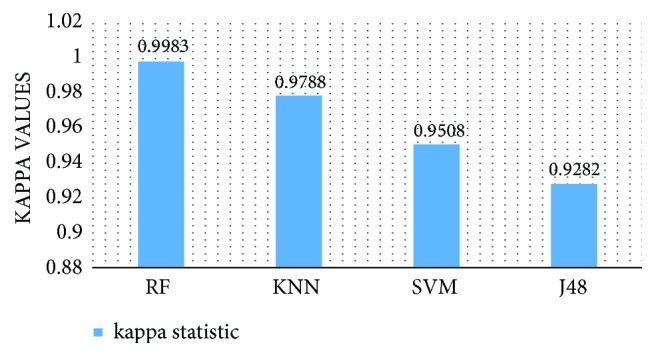
Kappa statistics values for the proposed classifiers.

In order to explore the best classifier for the DP_PCA features set, the performances of all four classifiers are compared in terms of kappa statistics and AUC.  Figure 11 presents the AUC for the RF,* k*-NN, SVM, and J48 classifiers for the DP_PCA features set, separately for each of five classes and their overall AUC as well. The AUC is used as a measure for assessing the classifier performance (e.g., a higher value of the area indicates better performance of the classifier). As can be seen in Figure 11, each of the four classifiers achieve high AUC close to 1 for each class (Set), and the RF classifier produces slightly higher AUC in each class comparing to the other three classifiers.  Figure 12 shows the performance of all reported classifiers in terms of kappa statistic. In this research, kappa statistics test is used to evaluate the consistency of the four classifiers: the RF,* k*-NN, SVM, and J48 on the DP_PCA features set. The kappa value (k) indicates the consistency of the classifier. The consistency is considered as mild if k<0.2, fair if 0.21 < k < 0.40, moderate if 0.41 < k < 0.60, good if 0.61 < k < 0.80, and excellent if k > 0.81. The maximum value of kappa is one which defines total consistency. As can be seen in Figure 12, the kappa values are very high (close to 1) for all four classifiers, and the RF classifier achieves the highest kappa value (K=0.998). From Figures 11 and 12, it is clear that the RF classifier yields better performance with the DP_PCA features set in the EEG signals classification than the other three classifiers. Therefore, The RF classifier is selected as the best classifier for the DP_PCA features set in epileptic EEG signal classification.

### 4.2. Comparison

Although there are many studies in the literature for epileptic EEG classification, most are restricted to the two-class classification problems dealing with the benchmark epileptic EEG data [32, 41–45]. Few studies have focused on the multiclass EEG signal classification [37, 39, 40, 46–48] (discussed in Section 2). To further evaluate the efficiency of the proposed method, a comparison of the proposed method with other six reported methods is provided.  Table 6 provides a comparative study between the proposed method and the three reference algorithms for the same benchmark epileptic EEG dataset. This table reports the overall classification performance of the five categories of EEG signals in terms of sensitivity, specificity, and the classification accuracy. The specificity is the complement of false positive rate (100 -* FPR*). The highest overall classification performances among all reported methods are highlighted in italic. From Table 6, it is observed that the proposed method achieves the highest performance in each statistical parameter of each class compared to the six reference methods. The* OCA* of the proposed method is 99.85% while they are 99.30%, 99.28%, 99.20%, 98.05%, 97.60%, and 93.63% for methods reported in [37, 39, 40, 46–48], respectively. These results indicate the proposed method outperforms all six referenced methods and improves the* OCA* by at least 0.55%.

## 5. Conclusion

This research introduces a new concept based on DP algorithm for extracting representative information from multicategory EEG signals data in the epileptic seizures identification. This study also investigated which machine leaning model (e.g., RF,* k*-NN, SVM and DT) is suitable for the proposed feature exaction method. The experimental results demonstrate that the proposed method is very effective and efficient for extracting distinguishable features from the epileptic EEG data. The high classification performances achieved by all reported classifiers confirm the consistency of the extracted features to detect epileptic EEG signals. The results show that the proposed RF classifier with the DP_PCA features yields the best overall performance as compared to the other classifiers. The results also indicate that our proposed method outperforms the existing methods for the same epileptic EEG database. To conclude, the DP algorithm is reliable for extracting the representative samples from the original EEG data and the RF with the proposed feature set is an effective classifier for the classification of multiclass EEG signals. A limitation of the current study is that the computational complexity of the proposed method grows as the EEG data size increases. Therefore, the proposed method may not work effectively and can take more time to process and classify very large EEG data. For future work, we plan to reduce the complexity of the proposed method by replacing the PCA and DP algorithms with existing low computational complexity techniques of PCA and DP.

## Figures and Tables

**Table 1:** Summary of the epileptic EEG data.

	Set A	Set B	Set C	Set D	Set E
Subjects	Five healthy subjects	Five healthy subjects	Five epileptic subjects	Five epileptic subjects	Five epileptic subjects
Patient's state	Awake and eyes open (normal)	Awake and eyes closed (normal)	Seizure-free (interictal)	Seizure-free (interictal)	Seizure activity (ictal)
Electrode type	Surface	Surface	Intracranial	Intracranial	Intracranial
Electrode placement	Inerenational 10-20 system	Inerenational 10-20 system	Epileptogenic zone	Epileptogenic zone	Epileptogenic zone
Number of channels	100	100	100	100	100
Time duration (s)	23.6	23.6	23.6	23.6	23.6

**Table 2:** The obtained value of *ϵ* for each *Segm* in each of the five classes.

	Segm 1	Segm 2	Segm 3	Segm 4
Set A	96.80	84.45	88.31	97.38
Set B	137	139.87	179.87	167.87
Set C	66.37	99.77	62.75	68.71
Set D	52.14	75.96	63.53	54.37
Set E	908.46	821.18	747.53	727.49

**Table 3:** The representative samples chosen by DP for each *Segm* in each of the five classes.

Sample size	Segm 1	Segm 2	Segm 3	Segm 4	Total
Set A	581 (1024)	612 (1024)	599 (1024)	562 (1025)	2354 (4096)
Set B	564 (1024)	583 (1024)	429 (1024)	432 (1025)	2008 (4096)
Set C	589 (1024)	408 (1024)	622 (1024)	618 (1025)	2237 (4096)
Set D	669 (1024)	489 (1024)	532 (1024)	708 (1025)	2398 (4096)
Set E	421 (1024)	438 (1024)	450 (1024)	449 (1025)	1758 (4096)

Note: in each cell, the number inside the parentheses is the *Segm* size (e.g., (1024) in the 1st cell) and the number outside of the parentheses is the sample size (e.g., 581 in the 1st cell) obtained by the DP.

**Table 4:** Classification results on the epileptic EEG data.

Classifiers	*Se* (mean ± standard deviation)					10-fold cross validation *OCA*
	Set A	Set B	Set C	Set D	Set E	(mean ± standard deviation)
RF	99.79± 0.36	99.85±0.24	99.96± 0.14	99.71± 0.34	100± 0.00	99.85± 0.13
*k*-NN	100± 0.00	99.75± 0.35	100± 0.00	100± 0.00	89.93±2.02	98.31± 0.32
SVM	100± 0.00	99.85± 0.34	100± 0.00	99.87± 0.20	76.56±3.53	96.11± 0.61
J48	95.80± 1.54	91.68± 1.31	93.52± 1.75	94.83± 1.33	95.39± 1.43	94.27± 0.78

**Table 5:** Obtained false positive rate (*FPR*) for each of the proposed classifiers.

Classifiers	Dataset	*FPR*(%)				
		Set A	Set B	Set C	Set D	Set E
RF	1-fold	0.00	0.11	0.23	0.00	0.00
	2-fold	0.00	0.00	0.00	0.00	0.00
	3-fold	0.00	0.23	0.12	0.12	0.00
	4-fold	0.00	0.00	0.23	0.00	0.00
	5-fold	0.00	0.00	0.12	0.00	0.00
	6-fold	0.00	0.00	0.00	0.00	0.00
	7-fold	0.12	0.00	0.12	0.00	0.00
	8-fold	0.24	0.00	0.00	0.00	0.00
	9-fold	0.00	0.00	0.23	0.00	0.00
	10-fold	0.00	0.00	0.00	0.00	0.00
	*Overall*	*0.04*±*0.08*	*0.03*±*0.08*	*0.11*±*0.10*	*0.01*±*0.04*	*0.00*±*0.00*

*k*-NN	1-fold	0.00	1.49	0.35	1.20	0.00
	2-fold	0.24	0.80	0.23	0.84	0.00
	3-fold	0.12	1.26	0.59	0.60	0.00
	4-fold	0.12	1.26	0.23	0.00	0.00
	5-fold	0.12	1.49	0.35	0.00	0.00
	6-fold	0.24	0.69	0.47	0.48	0.00
	7-fold	0.48	0.57	0.35	0.60	0.00
	8-fold	0.12	0.80	0.70	0.48	0.00
	9-fold	0.12	1.37	0.00	0.48	0.00
	10-fold	0.24	1.14	0.35	0.24	0.00
	*Overall*	*0.18*±*0.13*	*1.09*±*0.34*	*0.36*±*0.20*	*0.50*±*0.36*	*0.00*±*0.00*

SVM	1-fold	1.07	1.94	0.94	0.48	0.00
	2-fold	0.71	1.14	0.82	1.20	0.00
	3-fold	1.07	1.14	1.29	0.84	0.00
	4-fold	0.60	1.94	0.94	0.36	0.00
	5-fold	1.19	1.71	0.82	1.19	0.00
	6-fold	1.31	1.71	1.53	1.44	0.00
	7-fold	1.31	0.91	1.41	1.20	0.00
	8-fold	1.55	1.72	0.82	1.44	0.00
	9-fold	0.71	2.06	1.41	1.20	0.00
	10-fold	1.07	2.06	1.41	1.32	0.00
	*Overall*	*1.06*±*0.31*	*1.63*±*0.42*	*1.14*±*0.29*	*1.07*±*0.38*	*0.00*±*0.00*

J48	1-fold	0.59	2.17	2.00	2.03	0.44
	2-fold	1.43	1.60	1.06	1.56	0.00
	3-fold	1.66	1.60	2.00	2.63	0.22
	4-fold	1.19	0.69	2.82	2.03	0.44
	5-fold	1.19	2.40	2.46	1.91	0.22
	6-fold	1.19	1.14	2.47	0.96	0.78
	7-fold	1.07	2.51	2.12	1.68	0.11
	8-fold	0.83	1.60	2.00	1.20	0.11
	9-fold	1.79	1.83	2.35	1.20	0.44
	10-fold	0.83	2.52	2.70	2.28	0.11
	*Overall*	*1.18 *±*0.38*	*1.81 *±*0.61*	*2.20 *±*0.50*	*1.75 *±*0.53*	*0.29 *±*0.38*

**Table 6:** Comparison with the existing methods on epileptic EEG database.

Methods	Datasets	Statistical parameters (%)		
		Sensitivity (*Se*)	Specificity (*Sp*)	Overall classification accuracy (*OCA*)
Proposed Method	Set A	*99.79*	*99.96*	*99.85*
	Set B	*99.85*	*99.97*	
	Set C	*99.96*	*99.90*	
	Set D	*99.71*	*99.99*	
	Set E	*100.00*	*100.00*	

Emigdio et al. (2016) [37]	Set A	95.30	99.26	97.60
	Set B	96.83	98.69	
	Set C	97.23	99.53	
	Set D	98.40	99.42	
	Set E	99.28	99.80	

Murugavel and Ramakrishnan (2016) [48]	Set A	93.25	98.42	93.63
	Set B	93.63	98.36	
	Set C	94.00	98.16	
	Set D	94.13	97.17	
	Set E	93.13	99.54	

Ubeyli (2010) [47]	Set A	98.00	99.62	98.05
	Set B	98.25	99.53	
	Set C	98.13	99.37	
	Set D	98.00	99.15	
	Set E	97.88	99.87	

Ubeyli (2009) [46]	Set A	99.25	99.84	99.20
	Set B	99.13	99.81	
	Set C	99.25	99.72	
	Set D	99.38	99.62	
	Set E	99.00	100.00	

Ubeyli (2008) [39]	Set A	99.38	99.81	99.30
	Set B	99.25	99.87	
	Set C	99.13	99.78	
	Set D	99.50	99.65	
	Set E	99.25	100.00	

Guler and Ubeyli (2007) [40]	Set A	99.25	99.84	99.28
	Set B	99.38	99.84	
	Set C	99.25	99.75	
	Set D	99.38	99.65	
	Set E	99.13	100.00	

## Data Availability

The epileptic EEG data used to support the findings of this study are available from publicly available EEG database of Department of Epileptology, University of Bonn, Germany.
